# The care unit in nursing home research: Evidence in support of a definition

**DOI:** 10.1186/1471-2288-11-46

**Published:** 2011-04-14

**Authors:** Carole A Estabrooks, Debra G Morgan, Janet E Squires, Anne-Marie Boström, Susan E Slaughter, Greta G Cummings, Peter G Norton

**Affiliations:** 1 Faculty of Nursing, University of Alberta, Edmonton, Canada; 2 Canadian Centre for Health and Safety in Agriculture, University of Saskatchewan, Saskatoon, Saskatchewan, Canada; 3 Clinical Epidemiology Program, Ottawa Hospital Research Institute, Ottawa, Canada; 4 Department of Neurobiology, Care Sciences and Science, Division of Nursing, Karolinska Institute, Stockholm, Sweden; 5 Department of Family Medicine, University of Calgary, Calgary, Canada

## Abstract

**Background:**

Defining what constitutes a *resident care unit *in nursing home research is both a conceptual and practical challenge. The aim of this paper is to provide evidence in support of a definition of care unit in nursing homes by demonstrating: (1) its feasibility for use in data collection, (2) the acceptability of aggregating individual responses to the unit level, and (3) the benefit of including unit level data in explanatory models.

**Methods:**

An observational study design was used. Research (project) managers, healthcare aides, care managers, nursing home administrators and directors of care from thirty-six nursing homes in the Canadian prairie provinces of Alberta, Saskatchewan and Manitoba provided data for the study. A definition of care unit was developed and applied in data collection and analyses. A debriefing session was held with research managers to investigate their experiences with using the care unit definition. In addition, survey responses from 1258 healthcare aides in 25 of the 36 nursing homes in the study, that had more than one care unit, were analyzed using a multi-level modeling approach. Trained field workers administered the Alberta Context Tool (ACT), a 58-item self-report survey reflecting 10 organizational context concepts, to healthcare aides using computer assisted personal interviews. To assess the appropriateness of obtaining unit level scores, we assessed aggregation statistics (ICC(1), ICC(2), η^2^, and ω^2^), and to assess the value of using the definition of unit in explanatory models, we performed multi-level modeling.

**Results:**

In 10 of the 36 nursing homes, the care unit definition developed was used to align the survey data (for analytic purposes) to specific care units as designated by our definition, from that reported by the facility administrator. The aggregation statistics supported aggregating the healthcare aide responses on the ACT to the realigned unit level. Findings from the multi-level modeling further supported unit level aggregation. A significantly higher percentage of variance was explained in the ACT concepts at the unit level compared to the individual and/or nursing home levels.

**Conclusions:**

The statistical results support the use of our definition of care unit in nursing home research in the Canadian prairie provinces. Beyond research convenience however, the results also support the resident unit as an important Clinical Microsystem to which future interventions designed to improve resident quality of care and staff (healthcare aide) worklife should be targeted.

## Background

Mounting evidence points to the front-line clinical unit as the interface at which quality patient outcomes are achieved [1-3]. However, defining a unit can be a conceptual and practical challenge for researchers interested in studying the influence of clinical units on patient and resident outcomes. These clinical units are embedded in complex organizational configurations and organizations use the term 'unit' differently. The healthcare literature pertaining to the definition of a unit is sparse and the little literature that does exist points to problems defining a unit. Fennell et al [4] describe 'faded', 'blurred' and 'overlapping' unit boundaries where acute care hospitals provide long-term care, nursing homes provide sub-acute care, and physicians and nurses work in several clinics, specialty teams, hospitals or extended care units. Denis et al [5] speak of 'operating units' with emergent 'negotiated' organizational boundaries, which may not exist in the organizational chart, as the *de facto *elementary structures of a healthcare organization. These operating units with varying coordination mechanisms co-exist with formal structural boundaries. All of these features point to the need for a mutually agreed upon definition of 'unit' by setting (e.g., by long-term care, by acute care), so that researchers can, at minimum, compare interventions and resulting outcomes between organizations.

When the National Institute on Aging in the US funded the Special Care Units Initiative in the early 1990s, one of the first challenges identified by researchers in this field was to develop definitional clarity [6-8] and typologies of nursing home units [9,10]. Elements of the definitions and typologies of special care units included environment, program elements, characteristics of residents, and training of staff [6,9,10].

The literature on Clinical Microsystems also informs us with regard to defining front-line units. A Clinical Microsystem is "a small group of people who work together on a regular basis to provide care to discrete subpopulations of patients" [2] (2002:474); it shares aims, processes, information and outcomes. These Microsystems are argued to be the place where care is made; quality, safety, reliability, efficiency and innovation are made; and staff morale and patient satisfaction are made (http://dms.dartmouth.edu/cms/about/background/). Nelson et al [2] identify four essential elements of Microsystems, each of which informed our definition of a care unit: (1) a core team of healthcare professionals (i.e., group of individuals providing care in the collective for a cluster of residents), (2) a defined population to which care is provided, (3) an information environment to support the work of caregivers and patients, and (4) support staff, equipment, and a work environment. These units which are embedded in larger organizational structures evolve over time [11]. Studies from a variety of organizations including nursing homes in the US [12], Great Britain [13], and Scandinavia [14] have demonstrated that when these systems are identified and supported they can improve the quality, efficiency and safety of care processes.

The organizational literature identifies the ambiguity inherent in unit boundaries. Freeman [15] suggests that units are socially defined entities whose boundaries are 'permeable', shifting over space and time. He suggests that unit boundaries may be reasonably defined according to the membership of the unit, flow of information, and consequences of events for members of the unit. Kozlowski et al [16] attempt to resolve the confusion surrounding the definition of unit by distinguishing three basic types of unit properties: *global, shared *and *configural*. Global unit properties are relatively objective features of the unit including unit size and function. Shared unit properties originate with and are shared by unit members including experiences, attitudes, perceptions, values, cognitions and behaviors. Configural unit properties are the patterns or variability among members' contributions to the unit such as the diversity in demographic characteristics, personalities, social networks or behaviors.

In this paper we describe the process by which we reached a practical definition of unit in the nursing home context, and provide practical and statistical evidence that supports its use in health services research and its potential use in quality improvement activity in nursing homes.

### The Translating Research in Elder Care (TREC) Program

*Translating Research in Elder Care *(TREC) is a five year program of research seeking to identify modifiable features of context in residential long-term care (nursing home) settings associated with better resident and staff outcomes. It is situated in 36 nursing homes in the three Canadian prairie provinces (Alberta, Saskatchewan, Manitoba) and is described in detail elsewhere [17-19]. One of the core projects in TREC uses survey methods to identify modifiable elements of organizational context in the 36 nursing homes. Data are aggregated to define elements of organizational context such as leadership, culture and evaluation (expressed as feedback mechanisms) at the unit level. Hierarchical modeling techniques are then used to examine associations between organizational context and staff and resident outcomes.

We chose resident care units as the primary unit of analysis in the TREC program for three reasons. First, based on our and others' work in acute care hospitals and elsewhere [20,21], we believe that contextual elements such as culture are most identifiable and operate most actively at the level of the care unit. Second, based on the work in Clinical Microsystems [1,2,22,23] and our own previous work, we believe that these units are the primary locus of change and thus where interventions are most likely to lead to meaningful and sustained improvements. Finally, the use of care units as the primary unit of analysis increases the power of explanatory models, beyond for example either individual analyses or analyses involving entire facilities.

However, when we began data collection, our field teams reported that care units as defined by long-term care facilities appeared in some cases to have a different meaning than we had experienced in acute care settings. For example, in some facilities using alternative models of care (such as Eden), *houses *of 6-10 beds were called units but from a management and staffing perspective several of these would form a single traditional care unit of the type seen in acute care. Consequently, we undertook an iterative process with our field staff, to develop an explicit definition of 'care unit' that would serve the research purposes satisfactorily but still reflect meaningful organizational units. We aimed to develop a definition that was simple, practical in the field, reflected the 'care unit' as the locus of change, was applicable in the field to long-term care facilities, and importantly - where we could assign data from healthcare aides to specific care units designated by our definition. To develop this definition we reviewed the literature and had discussions with relevant stakeholders in the nursing home sector.

Our resulting definition of a unit was: A care unit is a geographic area in the long-term care facility with dedicated management. A care unit is characterized by:

• *A regular group of care providers (e.g., healthcare aides, LPNs, RNs ) who deliver the direct care and who work on the unit most of their shifts in the facility. The care providers may occasionally work across different units in the facility, especially on shift, but would normally work about 60% of their shifts on one unit*.

• *A care manager who is in charge of the unit overall. These supervisory tasks may stretch across several units for the supervision, e.g., registered nurses on night shift*.

• *A nurse who oversees the unit on a shift by shift basis. These supervisory tasks also may stretch across several units for the supervision, e.g., registered nurses on night shift*.

*Residents with similar care needs (e.g., dementia) are often grouped together on a care unit*.

### Aim

The aim of this paper is to provide evidence in support of a definition of care unit in nursing homes by demonstrating: (1) its feasibility for use in data collection, (2) the acceptability of aggregating individual responses to the unit level, and (3) the benefit of including unit level data in explanatory models.

## Methods

Data for the analysis reported in this paper are from data collected in TREC *Project 1*, and a debriefing session held with our provincial research managers who were responsible for data collection. The purpose of Project 1 is to monitor and examine organizational context over time in 36 nursing homes (30 urban, 6 rural). The data sets include nursing home and unit level data, provider (staff) level data, and resident level data. Urban nursing home selection was stratified (by healthcare region, owner operational model, and size) and used random sampling (see Table 1). The rural sample was a convenience sample intended for exploratory and descriptive purposes only and is not being used in primary TREC analyses; rural was defined using the Statistics Canada definition [http://www.statcan.gc.ca/pub/21-601-m/2002061/4224867-eng.pdf]. All nursing homes across the three Canadian prairie provinces that met our inclusion criteria [18] were eligible to participate. Ethics approvals were obtained from the research ethics boards of all investigator affiliated universities. Operational approvals were obtained from all relevant organizations.

**Table 1:** Nursing home characteristics (n = 36 nursing homes)

Nursing home ID	**Operation model**^**1**^	**Facility size**^**2**^	**No. of units**^**3**^	No. of healthcare aide responses	No. of healthcare aide responses/unit		Realignment of unit data based on TREC care unit definition
						
					Range	Mean (SD)	
**Urban Nursing Homes**							

G	Public	Small	1	32	NA	NA	Yes

B	Public	Small	2	32	12-20	16.00 (5.66)	No

O	Public	Small	5	46	7-11	9.20 (1.64)	Yes

D	Public	Medium	2	44	21-23	22.00 (1.41)	No

I	Public	Large	3	66	21-23	22.00 (1.00)	Yes

C	Public	Large	6	94	8-25	15.67 (6.22)	No

M	Public	Large	6	62	10-11	10.33 (0.52)	No

Q	Private	Small	1	14	NA	NA	No

E	Private	Small	1	27	NA	NA	Yes

Y	Private	Medium	3	37	11-13	12.33 (1.15)	No

P	Private	Large	2	31	14-17	15.50 (2.12)	No

V	Private	Large	3	53	12-21	17.67 (4.93)	No

L	Private	Large	4	73	15-22	18.25 (2.87)	Yes

K	Private	Large	5	77	13-17	15.40 (1.67)	No

A	Voluntary	Small	1	21	NA	NA	No

U	Voluntary	Small	1	15	NA	NA	Yes

AC	Voluntary	Small	2	30	12-18	15.00 (4.24)	No

H	Voluntary	Small	3	30	10-10	10.00 (0.00)	No

R	Voluntary	Medium	2	34	17-17	17.00 (0.00)	No

S	Voluntary	Medium	2	21	10-11	10.50 (0.71)	Yes

Z	Voluntary	Medium	2	30	13-17	15.00 (2.83)	No

AD	Voluntary	Medium	3	30	6-17	10.00 (6.08)	No

X	Voluntary	Medium	3	26	6-12	8.67 (3.06)	No

F	Voluntary	Medium	3	37	9-19	12.22 (5.77)	No

W	Voluntary	Medium	5	34	6-7	6.80 (0.45)	No

T	Voluntary	Large	3	38	9-17	12.67 (4.04)	No

AA	Voluntary	Large	4	49	8-14	12.25 (2.87)	No

N	Voluntary	Large	3	59	11-31	19.67(10.26)	Yes

AB	Voluntary	Large	5	61	9-15	12.20 (2.59)	No

J	Voluntary	Large	8	164	20-23	20.50 (1.07)	No

**Rural Nursing Homes**							

AE	Public	Small	2	25	NA	NA	No

AH	Public	Small	1	22	NA	NA	No

AI	Public	Small	1	17	NA	NA	Yes

AJ	Public	Small	1	9	NA	NA	No

AF	Public	Medium	3	36	9-18	12.00 (5.20)	No

AG	Voluntary	Small	1	13	NA	NA	Yes

^1^Operation model

• Private (for profit) facility = A facility in which the individual(s) or agency in control receives compensation other than wages, rent, or other expenses for the services they provide

• Public facility = A facility supported primarily through public funds, owned and operated by the local government

• Voluntary facility = A long-term care facility that is run by voluntary, cultural or religious organizations

^2^Facility size = number of long-term care beds; small: ≤ 80 beds, medium: 81-120 beds, large: > 120 beds

^3^No. of units = represents the number of units after realignment

Facility and unit level data (e.g., owner operational model, number of beds, number of units) were collected in short structured interviews by the research managers from nursing home administrators/Directors of Care and care managers respectively. Individual level data were collected from healthcare aides, nurses, allied providers, practice specialists, physicians and care managers using the *TREC survey*. Staff were recruited using volunteer, census sampling. The survey was administered to the healthcare aides (the dominant care provider group in Canadian nursing homes) using computer-assisted personal interviews (CAPI). The remaining staff groups completed the survey online. Resident level data are obtained from data routinely collected with the Resident Assessment Instrument/Minimum Data Set, version 2.0 (RAI-MDS 2.0), a comprehensive, standardized tool designed to assess residents' strengths, needs and potential risks in order to inform individualized care planning and monitoring (http://www.interrai.org). In this paper we report analyses that used data collected in year one (July 2008 - June 2009) of the TREC study from the following three sources: (1) facilities, (2) care units and (3) healthcare aides.

The TREC definition of care unit that we developed was applied to all 36 nursing homes. During year one, we tracked: (1) the research managers' assessments of unit structure as each nursing home and its units enrolled in the study, and (2) instances where we needed to 'realign' the unit boundaries *a posteriori *in our data. We also held a debriefing session with TREC project research managers following year one data collection to investigate their experiences with using the definition. A member of the TREC research team using a semi-structured interview guide facilitated the session, which was audio taped and transcribed.

### Measures - The TREC Survey

The TREC survey is a suite of instruments designed to measure organizational context, knowledge translation, and staff outcomes. The core of the survey is the Alberta Context Tool (ACT), a tool designed to measure organizational context in complex healthcare settings [24]. The ACT is premised on the *Promoting Action on Research Implementation in Health Services (PARiHS) framework *of research implementation which argues that successful implementation of research is a function of optimal levels of context, facilitation and robust evidence [25,26], and related literature [27-29]. Context refers to "...the environment or setting in which people receive healthcare services, or in the context of getting research evidence into practice, the environment or setting in which the proposed change is to be implemented" [30] (2004:299). According to the PARiHS framework, it is comprised of three core and interrelated dimensions: culture, leadership and evaluation. Expanded views of context, which also informed development of the ACT, can be found in related literature (e.g., [27-29,31,32]).

The healthcare aide version of the ACT reported in this paper contains 58 items reflecting 10 contextual concepts: culture, leadership, evaluation, social capital, formal interactions, informal interactions, structural and electronic resources, organizational slack-staff, organizational slack-space, and organizational slack-time. The survey was adapted for and piloted in the long-term care setting [33]. The ACT is described elsewhere [24] and a list of the 10 ACT concepts, their theoretical and operational definitions, are presented in Additional File 1.

### Analysis

To assess aim 1 (feasibility), we reviewed project documentation for all 36 nursing homes to identify instances where the TREC unit definition resulted in realignment of facility defined care units and reasons for this realignment. We also conducted a debriefing session with TREC research managers. Two members of the research team independently reviewed the transcript of the debriefing session to identify themes, which were subsequently refined through an iterative process of independent analysis followed by conference calls to discuss evolving themes and to reach consensus on the final analysis.

To assess aim 2 (the appropriateness of obtaining unit level scores) we assessed aggregation statistics. To assess aim 3 (the value of using the TREC care unit definition in planned explanatory models), we conducted multi-level modeling (Hierarchical Linear Modeling, HLM). We used Statistical Package for the Social Sciences for Windows (SPSS v. 18.0) [34] for these analyses. These latter analyses (aggregation and multi-level models) were done on responses from 25 of the 36 nursing homes (n = 89 units and n = 1243-1258 healthcare aide responses, depending on the ACT concept). We excluded the six rural nursing homes from the analysis reported in this paper because they were not part of the primary TREC nursing home sample. *Post hoc *assessment using the ACT confirmed differences in context between urban and rural nursing homes. In addition, the rural nursing homes tended to have only one unit. We also excluded from the analysis reported in this paper, the five urban nursing homes in which there was only one unit, as more than one unit is required to run the three-level models reported here.

We examined the aggregation properties for the healthcare aide data on the 10 ACT concepts at the unit and nursing home levels using four standard empirical aggregation indices: inter-class correlations (ICC(1) and ICC(2)), eta squared (η^2^), and omega squared (ω^2^). ICC(1) is an estimate of individual (healthcare aide) score variability about the subgroup mean; values greater than 0 (greater than 0.10 are preferred) indicate a degree of perceptual agreement among the healthcare aides about the mean values on the ACT concepts within each group (e.g., unit and/or nursing home) [35]. ICC(2) is an estimate of stability of aggregated data at the group level: values exceeding 0.60 justify aggregation [35]; η^2 ^is an indicator of effect size and contributes to the proportion of variance in the dependent variable accounted for by group membership [36]. ω^2 ^is a measure of the relative strength of the aggregated variable at the group level [37].

We then used multi-level modeling to assess whether aggregation of healthcare aide responses to the unit level (as defined by the TREC care unit definition) would lead to a higher amount of explained variance in the ACT concepts relative to no aggregation or aggregation to the nursing home level (i.e., would aggregation to the unit level increase the explanatory power in our models?). We ran 30 unconditional (null) models (3 models per ACT concept). The three models included two two-level models (unit and individual, and nursing home and individual) and one three-level model (nursing home, unit, and individual). We then compared the amount of variance explained among the three models, and assessed whether the variance at both the unit and nursing home levels was greater than 0. We used the Likelihood Ratio test to assess differences among the three models. As a final step, for the 10 ACT concepts, we assessed whether unit and nursing home variance were significantly greater than 0 and also the significance of between and within nursing home variance.

## Results

Characteristics of the 36 nursing homes are shown in Table 1.

### Study Aim #1: Feasibility of the definition

#### Realignment of units

We applied the TREC care unit definition in all of the nursing homes; in 10 (28%), we needed to use the definition to realign the unit bed structure (in our data) reported by the facility administrator for research purposes. In 9 of these 10 nursing homes this resulted in fewer care units than originally stated; in one nursing home it resulted in an increase in units (from 1 unit to 5) (see Table 2). The reasons for bed realignment are summarized in Table 2. Of the 10 nursing homes where data for unit beds were realigned, three had private, three had voluntary and four had public owner operational models; demonstrating that this realignment was required across all owner operational models.

**Table 2:** Nursing homes that required a realignment of unit beds (n = 10 nursing homes)

Nursing home ID	Realignment of unit data	Component of TREC definition that caused realignment		
		
		**1**^**1**^	**2**^**1**^	**3**^**1**^
E	3 units reconfigured to 1 unit	√	√	√

G	4 units reconfigured to 1 unit			√

I	9 units reconfigured to 3 units	√		

L	5 units reconfigured to 4 units	√	√	√

N	9 units reconfigured to 3 units	√		√

O	1 unit reconfigured to 5 units	√		√

S	4 units reconfigured to 2 units			√

U	3 units reconfigured to 1 unit	√		

AG	3 units reconfigured to 1 unit	√		√

AI	3 units reconfigured to 1 unit	√		√

^1^Component of TREC Definition:

1 = a regular group of care providers who deliver the direct care and who work on the unit most of their shifts in the facility

2 = a care manager who is in charge of the unit overall

3 = a nurse who oversees the unit on a shift by shift basis

#### The debriefing session

The three provincial research managers reported that the care unit definition was clear and that they used it to confirm the number of units in each nursing home prior to data collection. The number and names of units were required for programming the software for the CAPI interviews with the healthcare aides and for sampling, which was based on percentages of eligible aides by unit. Rather than asking the nursing home administrator or Director of Care to apply the definition, the research managers asked them about the number of units in their facility without providing a specific definition. The research managers then gathered information from the Directors of Care regarding assignment of front-line staff to specific geographic areas, how supervision was provided within and across shifts, and facility layout. This information was used to determine how the organizational definition of units provided by the Directors of Care fit with the TREC care unit definition. Once the definition had been developed and applied during year 1, the research managers did not need to revisit it in year 2.

The research managers did not discuss the unit definition directly with the healthcare aides although they did sometimes ask the healthcare aides questions about staffing assignments and supervision patterns to assess the fit between the care unit definition and the information provided by the Director of Care. The research managers noted that in some nursing homes the Director of Care and healthcare aides would use a different name for a given unit (e.g., 'dementia unit' rather than the formal unit name).

### Study Aim #2: Aggregation of individual responses to the unit

The aggregation statistics (Table 3) generally supported the acceptability of aggregating the healthcare aides' responses on the ACT (survey) concepts at the level of the care unit. The range of ICC(1) values (greater than 0.00 for all 10 ACT concepts, and greater than 0.10 for four concepts) indicated a degree of perceptual agreement among the healthcare aides within care units about the mean values on the ACT concepts. The ICC(2) values were high (greater than 0.60 for five concepts), indicating reliability of the data when aggregated to the care unit. The relative effect sizes (indicated by η^2 ^and ω^2^) were, on average, low to moderate for the ACT concepts, suggesting as expected that, as the healthcare aide responses on the ACT concepts were aggregated, our ability to assign the same meaning to the concept at the care unit level as at the individual level, decreased. These four standard indices were also assessed at the nursing home level; however, findings were stronger for aggregation at the care unit level (see Table 3).

**Table 3:** Aggregation measures (n = 25 nursing homes)

Dimension	F	BMS	WMS	ICC1	ICC2	η2	ω2	PROB
**UNIT LEVEL AGGREGATION**								

Leadership	2.1733	0.7596	0.3495	**0.0768**	0.5399	0.1417	0.0765	0.0000

Culture	2.9261	0.7410	0.2533	**0.1201**	0.6582	0.1814	0.1193	0.0000

Evaluation	2.3907	0.7391	0.3092	**0.0897**	0.5817	0.1540	0.0895	0.0000

Social Capital	1.7570	0.4074	0.2319	**0.0509**	0.4308	0.1181	0.0508	0.0000

Formal Interactions	1.5716	0.8838	0.5624	**0.0389**	0.3637	0.1064	0.0387	0.0009

Informal Interactions	1.3070	3.3072	2.5304	**0.0213**	0.2349	0.0906	0.0213	0.0342

Structural and Electronic Resources	2.5201	6.8517	2.7189	**0.0972**	0.6032	0.1612	0.0972	0.0000

OS-staff	7.8907	7.3179	0.9274	**0.3281**	0.8733	0.3728	0.3254	0.0000

OS-space	7.7267	5.4560	0.7061	**0.3228**	0.8706	0.3685	0.3207	0.0000

OS-time	4.9345	2.8298	0.5735	**0.2180**	0.7973	0.2724	0.2170	0.0000

**NURSING HOME LEVEL AGGREGATION**								

Leadership	3.7238	1.3391	0.3596	**0.0521**	0.7315	0.0682	0.0498	0.0000

Culture	7.0012	1.8055	0.2579	**0.1079**	0.8572	0.1205	0.1032	0.0000

Evaluation	4.5091	1.4341	0.3181	**0.0661**	0.7782	0.0815	0.0634	0.0000

Social Capital	3.2422	0.7592	0.2342	**0.0432**	0.6916	0.0600	0.0415	0.0000

Formal Interactions	2.6004	1.4760	0.5676	**0.0313**	0.6154	0.0485	0.0298	0.0000

Informal Interactions	2.0836	5.2765	2.5324	**0.0214**	0.5201	0.0394	0.0205	0.0017

Structural and Electronic Resources	5.8401	16.0841	2.7541	**0.0889**	0.8288	0.1032	0.0855	0.0000

OS-staff	23.9814	22.9152	0.9555	**0.3166**	0.9583	0.3184	0.3050	0.0000

OS-space	22.1040	16.3660	0.7404	**0.2985**	0.9548	0.3015	0.2877	0.0000

OS-time	14.6247	8.4892	0.5805	**0.2155**	0.9316	0.2229	0.2075	0.0000

F = test statistic from one-way analysis of variance (ANOVA)

The source table from ANOVA was used to calculate Interclass correlation 1 (ICC 1), Interclass correlation 2 (ICC 2), Eta Square (η^2^), and Omega Square (ω^2^) as follows:

1. ICC(1) = (BMS - WMS)/(BMS + [K - 1] WMS), where BMS is the between-group mean square, WMS is the within-group mean square, and K is the number of subjects per group. The average K for unequal group size was calculated as K = (1/[N - 1]) (ΣK - [ΣK^2^/ΣK]);

2. ICC(2) = (BMS - WMS)/BMS;

3. η2 = SSB/SST, where SSB is the sum of squares between groups and SST is the sum of squares total; and

4. ω^2 ^= (SSB - [N - 1] WMS)/(SST + WMS).

### Study Aim #3: Adding value to explanatory models

The percentages of total variance (for each ACT concept) that were explained at the unit and nursing home levels are summarized in Table 4. We also calculated the amount of explained variance gained for each ACT concept by aggregating responses to higher levels. For each ACT concept, a higher percentage of variance, at statistically significant levels, was explained by aggregating the healthcare aide responses to the care unit and also to the nursing home, when compared to maintaining scores at the individual level. The only exception was informal interactions where the increase in variance at the unit (over individual level) was not statistically significant.

**Table 4:** Results of multi-level analysis (analysis of explained variance for 10 ACT concepts at unit and nursing home levels, n = 25 nursing homes)

**ACT Concept**^**1**^	**% Variance that can be explained by**:			**% of explained variance gained by**:		H0: Variance = 0 (Ha: Variance > 0) (P value)	
	**Unit **^**2**^	**Nursing Home**^**3**^	**Unit + Nursing Home**^**4**^	**Aggregating to nursing home**^**5**^	**Aggregating to unit**^**6**^	**Nursing Home = 0 (3 level model)**	**Unit = 0 (2 level model)**

Leadership	7.73%*	6.08%*	8.49%*	0.77%	2.41%	**0.0130**	**0.0002**

Culture	11.84%*	10.59%*	11.79%*	-0.05%	1.20%	0.1230	**<0.0001**

Evaluation	8.98%*	6.42%*	8.34%*	-0.64%	1.91%	0.0726	**0.0001**

Social Capital	5.08%*	4.18%*	4.90%*	-0.18%	0.72%	0.2399	**0.0030**

Formal Interactions	3.63%*	2.92%*	3.73%*	0.10%	0.81%	0.2171	**0.0140**

Informal Interactions	2.15%	1.97%*	2.36%*	0.21%	0.38%	0.3284	0.0557

Resources	9.74%*	10.20%*	11.36%*	1.62%	1.16%	0.1006	**<0.0001**

OS-staff	32.89%*	28.84%*	30.92%*	-1.97%	2.09%	**0.0251**	**<0.0001**

OS-space	34.45%*	31.74%*	35.05%*	0.60%	3.30%	**0.0037**	**<0.0001**

OS-time	21.91%*	19.76%*	20.48%*	-1.42%	0.72%	0.2212	**<0.0001**

^1 ^n values. Facility (n = 25), Unit (n = 89), Healthcare Aide responses (n = 1243 to 1250 depending on the ACT concept: leadership n = 1247, culture n = 1251, evaluation n = 1245, social capital n = 1244, formal interactions n = 1250, informal interactions n = 1244, structural and electronic resources n = 1243, OS-staff n = 1257, OS-space n = 1254, OS-time n = 1249)

^2 ^2 level model (unit, individual). % of total variance that can be explained by the unit level

^3 ^2 level model (nursing home, individual) % of total variance that can be explained by the nursing home

^4 ^3 level model (nursing home, unit, individual) % of total variance that can be explained by unit + nursing home

^5 ^variance gained by adding nursing home (% unit + nursing home explained variance - % unit explained variance)

^6 ^variance gained by adding unit (% unit + nursing home explained variance - % nursing home explained variance)

* p < 0.05 compared to individual only level (Likelihood Ratio test)

We also examined the amount of explained variance *gained *by moving from the individual level (i.e., the level where data collection occurred) to the care unit and nursing home levels. For 9 of the 10 ACT concepts, the amount of explained variance gained was higher at the care unit level than at the nursing home level (Table 4). We also assessed the p-values associated with the null hypotheses (that variance = 0) at both care unit and nursing home levels (Table 4). At the nursing home level, we can reject the null hypothesis that nursing home variance = 0 at p < 0.05 for only three concepts: leadership, organizational slack-staff, and organizational slack-space. However, at the unit level we can reject the null hypothesis that unit variance = 0 at p < 0.05 for 9 of the 10 ACT concepts; the p-value of one concept (informal interactions) was 0.0557. These findings suggest there is *benefit to explaining variance in organizational context when responses of healthcare aides are aggregated to the level of the care unit *in nursing home research.

As a final assessment, we examined variance on the 10 ACT concepts among the 25 nursing homes and also between care units within each nursing home. We found statistically significant between-nursing home variance for all 10 ACT concepts. Statistically significant within-nursing home variation was also found for 6 of the 10 ACT concepts: leadership, culture, evaluation, organizational slack-staff, organizational slack-space, and structural and electronic resources.

## Discussion

As the existing demographic patterns in our populations continue to shift us to higher proportions of old and very old adults, we will see greater numbers of these individuals experiencing dementia - and consequently greater numbers requiring nursing home placement [38,39], especially in the later stages of dementia. With these shifts will come increasing need and pressure for investigations to contribute meaningfully to both quality of care and quality of life for this group of frail and vulnerable seniors living in residential long-term care (nursing home) settings. The findings reported in this paper contribute to our understanding of the design of such studies first, by offering a workable definition of care unit; second, by providing early evidence using one tool (the ACT) that aggregation to *care units *is defensible and in fact, offering superior performance to that of nursing home level aggregation; and third, by demonstrating that better explanatory power is possible when appropriately using care unit level variables in models. These findings together with the clear alignment of our definition with that of Clinical Microsystems lead us to believe that we are defining emerging Microsystems. As we indicated in our introduction there is promising evidence that the definition and support of such entities may lead to significant gains in quality, efficiency and safety of care delivery in the sector. Additional investigation is required to understand both the management and cost implication of integrating our definition into the management models for the nursing home sector.

### Study Aim #1: Feasibility of the definition

The definition we developed, and subsequently used, enabled the fieldworkers to ascertain the number of care units within each nursing home with increasing independence as the study progressed. An important implication is that future studies would benefit from careful *a priori *consensus on a care unit definition and equally careful attention to both operationalizing the definition and training data collection personnel in its use. Finally, validation with relevant stakeholders is an important step.

While we completed the statistical work in this paper on a subset of 25 nursing homes, the definition was applicable and was used in all 36 nursing homes in the study. In 10 of the 36 (28%) nursing homes, application of the definition resulted in a different realignment than would otherwise have occurred. The realignment of data in these 10 nursing homes was due to a mixture of the different components of the definition as reflected in Table 2. In the majority of cases the adjustments resulted from: (1) assessing the group (i.e., healthcare aides) providing care for residents more carefully specifically regarding 'working together consistently', and (2) whether a professional nurse (registered nurse or licensed practical nurse) was present on all shifts. As a result we usually merged more units into fewer units. This is consistent with Kozlowski's discussion of *shared *units [16] and reflects the lack of sharp boundaries as identified by Fennel [4] and Denis [5].

### Study Aim #2: Aggregation of individual responses to the unit

Aggregation of individual data to higher organizational levels as occurs in educational research is an important design and methodological issue that has received relatively little attention in health services and long-term care literature. While preferable, direct measurement of a phenomenon (i.e., bed size or owner/operator model) is not possible for some concepts (e.g., unit culture), and investigators must use responses obtained from individuals if they wish to include such concepts in their studies. There are no hard and fast rules that guide selection of which individuals should provide the responses, the numbers of responses that are required to achieve stable estimates, or for the best methods to combine the scores to reach an aggregate score [40,41]. We argued in this study that the most relevant responses were those of the group providing the vast majority of the daily, face-to-face care for the residents (i.e., healthcare aides). We argued this because we are interested in modifiable elements of context that directly affect resident care. Others, usually from the business environment, have argued for responses from senior executive team members [42,43]. Our findings support aggregating the *healthcare aides *responses to obtain unit level scores in nursing homes.

### Study Aim #3: Adding value to explanatory models

We achieved greater explained variance (and thus more explanatory power) at the care unit vs. the nursing home level (Table 4), and the hypothesis that variance was not zero was consistently supported at the care unit over the nursing home level, with the context concepts included in the ACT. While this result is important for our planned modeling activity, we believe it also serves as a preliminary indication that the TREC definition of care unit may be important for those who manage nursing homes. In particular we have shown that context in the PARiHS framework, as measured by the ACT, appears to be primarily a unit level construct. Drawing upon the premises of the PARiHS framework and with the *Clinical Microsystems *approach, our results suggest that the care unit is where practice improvement must occur [1,2,22,23]. Future research will be necessary to demonstrate more conclusively that care units of the type we have defined are the major units of change in nursing homes and should be the focus of quality improvement activity.

### Limitations

First, the findings should not be generalized at this time beyond nursing homes in the three Canadian prairie provinces. Our findings may not be applicable to, for example, Ontario's *complex continuing care *settings that typically have higher acuity levels or to US nursing homes where funding structures are different and there may be greater overlap between acute and long-term care. Second, we used healthcare aide responses to obtain our unit level scores. Based on work we have done in pediatric acute care settings [24] we know that aggregating using different groups of staff (e.g., registered nurses, allied health professionals) sometimes produces different results, depending on how their work is structured. One can expect either the care unit or the facility (hospital or nursing home) aggregation to be favoured depending on the work structures of these staff. The choice of which individual responses to use when aggregating should be theoretically driven and informed by a substantive knowledge of the clinical environment in question.

## Conclusions

In this paper we described the application of a practical working definition of care unit to study data from healthcare aides working in nursing homes. Our findings further indicate that context in the PARiHS framework, as measured by the ACT in the TREC survey, is a unit level construct. This has important implications for future research and quality improvement activity in nursing homes. We have provided early evidence that the ACT is a practical and sufficiently robust tool to warrant further use and assessment in research in this sector. The ACT was designed, and its elements worded, to elicit self-report focused specifically on the unit on which respondents work, and is able to discriminate among care units on key dimensions of modifiable organizational context.

## Abbreviations

TREC: Translating Research in Elder Care; ACT: Alberta Context Tool; LPN: Licensed practical nurse; RN: Registered nurse; CAPI: Computer-assisted personal interviews.

## Competing interests

The authors declare that they have no competing interests.

## Authors' contributions

CAE, PGN, GGC, and DGM participated in conceptualizing the TREC program and in secured the grant that provided its funding. CAE, PGN, GGC, and JES participated in conceptualizing project 1 and CAE, PGN, GGC, DGM, and JES in data collection. CAE led the statistical analytic design presented in the manuscript. JES assisted in performing and in interpreting the statistical analysis. DGM and AMB performed the analysis of the debriefing session. All authors contributed to drafting the manuscript and approved the final version.

## Pre-publication history

The pre-publication history for this paper can be accessed here:

http://www.biomedcentral.com/1471-2288/11/46/prepub

## Supplementary Material

Additional File 1 **ACT Concepts and Definitions (Long-Term Care, Healthcare Aide version)**. *This file contains a theoretical definition, description of operationalization, and a sample item for each the 10 ACT concepts*. Click here for file
