# Supplementary material for: The care unit in nursing home research: Evidence in support of a definition
Source: BMC Med Res Methodol. 2011 Apr 14;11:46. doi: 10.1186/1471-2288-11-46 (PMC3098823; doi:10.1186/1471-2288-11-46)
Supplement: Additional File 1 — ACT Concepts and Definitions (Long-Term Care, Healthcare Aide version). This file contains a theoretical definition, description of operationalization, and a sample item for each the 10 ACT concepts. [file 1471-2288-11-46-S1.DOC]

**Additional File 1. ACT Concepts and Definitions** (Long-Term Care, Healthcare Aide version)

| **Concept** | **Definition** | **Operationalization** | **Sample item** |
| --- | --- | --- | --- |
| Leadership | The actions of formal leaders in an organization (unit) to influence change and excellence in practice, items generally reflect emotionally intelligent leadership | Six items scored on a five point Likert agreement scale as follows:  1-strongly disagree  2-disagree  3-neither agree or disagree  4-agree  5-strongly agree | The leader calmly handles stressful situations |
| Culture | The way that ‘we do things’ in our organizations and work units; items generally reflect a supportive work culture | Six items scored on a five point Likert agreement scale as follows:  1-strongly disagree  2-disagree  3-neither agree or disagree  4-agree  5-strongly agree | My organization effectively balances best practice and productivity |
| Evaluation | The process of using data to assess group/team performance and to achieve outcomes in organizations or units (i.e., feedback processes) | Six items scored on a five point Likert agreement scale as follows:  1-strongly disagree  2-disagree  3-neither agree or disagree  4-agree  5-strongly agree | Our team routinely monitors our performance with respect to the action plans |
| Social Capital | The stock of active connections among people. These connections are of three types: bonding, bridging, and linking | Six items scored on a five point Likert agreement scale as follows:  1-strongly disagree  2-disagree  3-neither agree or disagree  4-agree  5-strongly agree | People in the group share information with others in the group |
| Informal Interactions | Informal exchanges that occur between individuals working within an organization (unit) that can promote the transfer of knowledge | Nine items scored on a five point Likert frequency scale as follows:  1-never  2-rarely  3-ocasionally  4-frequently  5-almost always | How often did you talk with people in the following roles or situations about resident care?  - Quality improvement representative/specialist? |
| Formal  Interactions | Formal exchanges that occur between individuals working within an organization (unit) through scheduled activities that can promote the transfer of knowledge | Four items scored on a five point Likert frequency scale as follows:  1-never  2-rarely  3-ocasionally  4-frequently  5-almost always | How often did you participate in the following?  - Team meetings about residents? |
| Structural/ Electronic Resources | The structural and electronic elements of an organization (unit) that facilitate the ability to assess and use knowledge | Eleven items scored on a five point Likert frequency scale (plus not available option) as follows:  1-never  2-rarely  3-ocasionally  4-frequently  5-almost always  6-not available | How often did you use the following while at work?  - A library? |
| Organizational Slack (3 concepts) | The cushion of actual or potential resources which allows an organization (unit) to adapt successfully to internal pressures for adjustments or to external pressures for changes | Ten items scored on a five point Likert agreement scale as follows:  1-strongly disagree  2-disagree  3-neither agree or disagree  4-agree  5-strongly agree | We have enough staff to get the *necessary w*ork done. |
| Staff |  |
| Space |  |
| Time |  |
